# Indocyanine green fluorescence-guided intraoperative detection of peritoneal carcinomatosis: systematic review

**DOI:** 10.1186/s12893-020-00821-9

**Published:** 2020-07-17

**Authors:** Gian Luca Baiocchi, Federico Gheza, Sarah Molfino, Luca Arru, Marco Vaira, Simone Giacopuzzi

**Affiliations:** 1 Department of Clinical and Experimental Sciences, University of Brescia, Brescia, Italy; 2 Oncoteam Peritoneal Surface Malignancies, SICO (Italian Society of Surgical Oncology), Brescia, Italy; 3 Centre Hospitalier de Luxembourg, Service de Chirurgie Generale, Luxemborg City, Luxembourg; 4 Surgical Oncology Unit, Candiolo Cancer Institute, FPO - IRCCS, Str. Prov.le 142, km. 3,95, 10060 Candiolo, TO Italy; 5 Department of Surgery, General and Upper G.I. Surgery Division, University of Verona, Verona, Italy

**Keywords:** Indocyanine green, Fluorescence, Peritoneal carcinomatosis, Colorectal cancer, Ovarian cancer, Gastric cancer

## Abstract

**Background:**

To review the available clinical data about the value of Indocyanine Green (ICG) fluorescence imaging for intraoperative detection of peritoneal carcinomatosis.

**Methods:**

We conducted a systematic review, according to the PRISMA guidelines, for clinical series investigating the possible role of ICG fluorescence imaging in detecting peritoneal carcinomatosis during surgical treatment of abdominal malignancies. With the aim to analyze actual application in the daily clinical practice, papers including trials with fluorophores other than ICG, in vitro and animals series were excluded. Data on patients and cancer features, timing, dose and modality of ICG administration, sensitivity, specificity and accuracy of fluorescence diagnosis of peritoneal nodules were extracted and analyzed.

**Results:**

Out of 192 screened papers, we finally retrieved 7 series reporting ICG-guided detection of peritoneal carcinomatosis. Two papers reported the same cases, thus only 6 series were analyzed, for a total of 71 patients and 353 peritoneal nodules. The investigated tumors were colorectal carcinomas in 28 cases, hepatocellular carcinoma in 16 cases, ovarian cancer in 26 cases and endometrial cancer in 1 case. In all but 4 cases, the clinical setting was an elective intervention in patients known as having peritoneal carcinomatosis. No series reported a laparoscopic procedure. Technical data of ICG management were consistent across the studies. Overall, 353 lesions were harvested and singularly evaluated. Sensitivity varied from 72.4 to 100%, specificity from 54.2 to 100%. Two series reported that planned intervention changed in 25 and 29% of patients, respectively.

**Conclusion:**

Indocyanine Green based fluorescence of peritoneal carcinomatosis is a promising intraoperative tool for detection and characterization of peritoneal nodules in patients with colorectal, hepatocellular, ovarian carcinomas. Further prospective studies are needed to fix its actual diagnostic value on these and other abdominal malignancies with frequent spread to peritoneum.

## Background

Peritoneal carcinomatosis represents a challenging localization of abdominal tumors. Detecting the presence of peritoneal involvement at an early stage could improve oncological results [1], both customizing the therapeutic path and enhancing the possibilities of surgical treatment. The results of cytoreductive surgery, possibly associated with hyper-thermic intraperitoneal chemotherapy (HIPEC), are directly dependent on the completeness of the cytoreduction, and this in turn is directly dependent on the size and number of the nodules.

Available preoperative staging systems have low sensitivity in detecting peritoneal carcinomatosis smaller than 5 mm [2, 3]; furthermore, even the visual inspection during staging laparoscopy or laparotomy may miss some small nodules, especially in areas needing bowel mobilization such as pelvis and mesentery. Often, a negative visual inspection is followed by the appearance of peritoneal localization disease at a short distance of time, demonstrating the substantial inadequacy of simple visualization with the naked eye in the early phases of peritoneal implant.

In the search for technologies going beyond these limits, the ability of indocyanine green (ICG) fluorescence to light up peritoneal nodules was assessed [4]; some studies have established that ICG binds to cancer cells, and modern cameras detect even the smallest quantities of fluorescence. If the above were confirmed, considering the widespread availability and ease of use of ICG fluorescence, this new technique would become widely used for studying the peritoneum. This systematic review analyzes available clinical trials including patients studied for peritoneal carcinosis with intravenous indocyanine green injection.

## Methods

We conducted a systematic review according to the guidelines set out in the Preferred Reporting in Systematic Review & Meta-Analysis (PRISMA) [5] and Assessing the methodological quality of systematic reviews (AMSTAR)) checklists. Embase, MEDLINE (PubMed), Cochrane library, Google Scholar Medline and Web of Knowledge databases were searched for, using MeSH terms and free text key words for clinical series investigating the role of ICG fluorescence imaging in detecting peritoneal carcinomatosis. The website www.clinicaltrials.gov was also visited.

Search was performed in April 2020 by 1 Author (LB); papers published in the period 2000–2020 were considered. No language limitations were provided. MeSH terms were Peritoneal Neoplasms [C04.588.033.513, C04.588.033.513, C04.588.274.780, C06.301.780, C06.844.620], Abdominal Neoplasms [C04.588.033], Peritoneal Diseases [C06.844], Indocyanine Green [D03.633.100.473.400], Fluorescence [G01.590.540.665.500], Neoplasm Metastasis [C04.697.650, C23.550.727.650]. Free Key words for search were “indocyanine green”, *“carcinomatosis”, “peritoneum”, “fluorescence”.* Papers providing the utilization of fluorophores other than ICG were excluded. Papers including in vitro and animals experiments were excluded. Case reports with < 2 cases were excluded. Studies in which ICG was linked to any molecular probe specific for cancer cells were also excluded. Three reviewers (SM, FG and LA) independently revised the literature search, evaluated relevant articles in full text, further searched for other articles included in the references, and achieved consensus on duplicate; they extracted the following data from studies: patients and cancer features, timing, dose and modality of ICG administration, sensitivity, specificity and accuracy of fluorescence diagnosis of peritoneal nodules. We analyzed the data and reported the results in tables and text.

Descriptive data (patients number, cancer type, fluorescence technology, timing and dosage of ICG injection) were reported in tables. Diagnostic values are reported in terms of sensitivity and specificity: sensitivity was computed as the number of true positive peritoneal nodules / (number of true positive + false negative) × 100%; specificity was computed as the number of true negative peritoneal nodules / (number of true negative + false positive) × 100%. Histopathological analysis was the gold standard in all the papers. Due to the limited number of retrieved papers, and the absence of prospective randomized or retrospective controlled trial, a robust technique for assessing the risk of bias was not implemented. However, the likely impact of selection bias on the results is analyzed and discussed. This study received no funding.

## Results

**Fig. 1:**
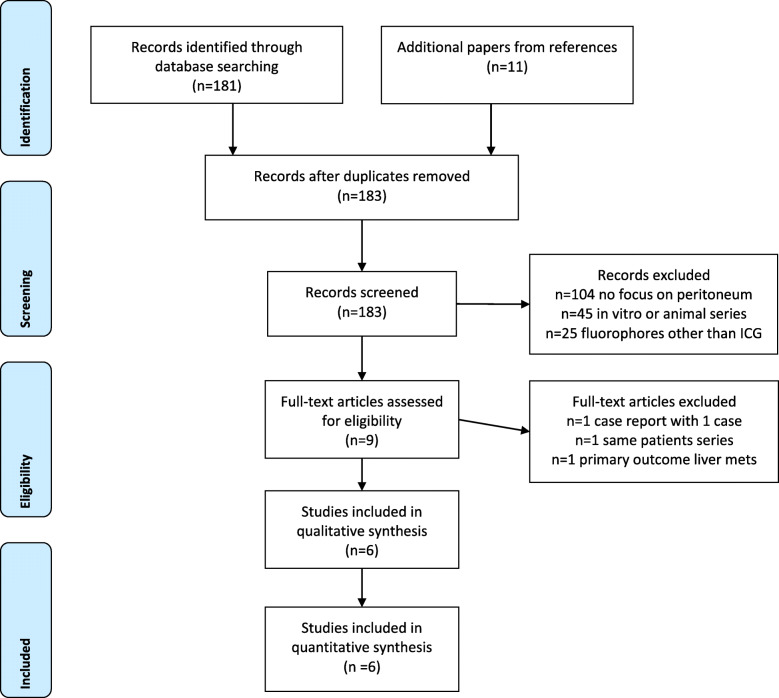
Flow diagram of studies selection. Articles retrieval strategy, according to the Preferred Items for Reporting of Systematic Reviews and Meta-Analyses guidelines

The process of study selection is reported in Fig. 1. Out of 192 screened papers, we finally retrieved 9 series reporting ICG-guided detection of peritoneal carcinomatosis. All the series were published in the period 2015–2018. A case report including only 1 patient with peritoneal recurrence of ruptured HCC was analyzed and excluded, according to selection criteria [6]. One series was further not analyzed because the primary outcome was liver metastases detection [7]. Two papers reported the same series [8, 9] (Clinical Trial Number 01982227), thus 6 series were finally analyzed [9–14].

Overall, 71 patients were included (Table 1). The primary tumor was colorectal carcinomas in 28 cases, hepatocellular carcinoma in 16 cases, ovarian cancer in 26 cases and endometrial cancer in 1 case. In all but 4 cases, the clinical setting was an elective intervention in patients known as having peritoneal carcinomatosis, and undergoing cytoreductive surgery, eventually associated with HIPEC. Only in 4 cases (3 ovarian cancer, 1 endometrium cancer) the peritoneal carcinomatosis was unknown before the intervention. No series reported a laparoscopic procedure.

**Table 1:** Analyzed series of peritoneal carcinomatosis assessment by ICG fluorescence

Author	Year	Pts number	Tumor	Setting	Surgical approach
**Barabino** [9]	*2016*	*10*	*CRC*	*Known PC, undergoing CRS + HIPEC*	*Open*
**Liberale** [10]	*2016*	*14*	*CRC*	*Known PC, undergoing CRS + HIPEC*	*Open*
**Lieto** [11]	*2018*	*4*	*CRC*	*Known PC, undergoing CRS + HIPEC*	*Open*
**Satou** [12]	*2012*	*16*	*HCC*	*Known extrahepatic HCC recurrence*	*Open*
**Tummers** [13]	*2015*	*7*	*Ovarian (6)* *endometrium (1)*	*4 Staging, 3 Radical surgery, unknown PC*	*Open*
**Veys** [14]	*2018*	*20*	*Ovarian*	*Known PC, undergoing surgery*	*Open*

*CRC* colorectal cancer, *HCC* hepatocellular carcinoma, *HIPEC* Hyperthermic Intra PEritoneal Chemotherapy

Technical data of ICG management are reported in Table 2. The injection time varied from 24 h before surgery to intra-operative injection. Dosing was consistently 0.25 mg/kg in 5 series; in the remaining 2 series, 0.5 mg/kg and 20 mg were given, respectively. Some 4 different camera systems were utilized, all with hand-easy to use camera allowing exploration of peritoneum without the need for handling the laparoscopic instruments during open intervention.

**Table 2:** Timing, dosing, route of administration and technological supplies for peritoneal carcinomatosis assessment by ICG fluorescence

Author	Time (h before incision)	Dose	Route for ICG administration	Camera system
**Barabino** [9]	*24 h*	*0,25 mg/kg*	*Intravenous*	*Fluostick (Fluopics, Grenoble, France)*
**Liberale** [10]	*0 h (assessment after 5′-40′)*	*0,25 mg/kg*	*Intravenous*	*Photodynamic Eye, PDE (Hamamatsu Photonics, Japan)*
**Lieto** [11]	*0 h (assessment after 20′ (10′-30′)*	*0,25 mg/kg*	*Intravenous*	*Fluobeam (Fluoptics Imaging Inc, Cambridge, MA, USA),*
**Satou** [12]	*1–5 d (1 pts 24 d)*	*0, 5 mg/kg*	*Intravenous*	*PDE (Hamamatsu Photonics, Hamamatsu, Japan)*
**Tummers** [13]	*0 h (assessment after median 37′, max 141′)*	*20 mg*	*Intravenous*	*Mini-Fluorescence-Assisted Resection and Exploration (Mini-FLARE)*
**Veys** [14]	*0 h (assessment after 5′-360′, mean 60′)*	*0, 25 mg/kg*	*Intravenous*	*Photodynamic Eye, PDE (Hamamatsu Photonics, Japan)*

Table 3 reports the clinical results. Overall, 353 lesions were singularly analyzed and evaluated as being ICG+ or ICG-, and malignant or benign. Sensitivity varied from 72.4 to 100%, specificity from 54.2 to 100%; the 2 most representative series (88 and 102 nodules analyzed, respectively) reported 72.4 and 72.6% sensitivity, and 60.0 and 54.2% specificity, respectively. Two series described having changed the planned intervention in 25 and 29% of patients, respectively. Two series analyzed subgroup of patients, with particular reference to mucinous colorectal cancers [10] and to ovarian cancers having been trated with neoadiuvant chemotherapy with good clinical response (the so-called “peritoneal scars”) [14]. Both subgroups showed a reduced positive predictive value. In 3 series a quantitative assessment of ICG fluorescence was performed [10, 13, 14], by calculation of the tumor-to-background ratio (TBR). In the remaining series, only the absolute visual evaluation (ICG + versus ICG -) was provided. The TBR values for peritoneal caricinomatosis nodules were comprised between 1.8 and 2.0.

**Table 3:** Peritonel carcinomatosis assessment by ICG fluorescence. Results reported by analyzed series

Author	Lesions analyzed	Sensitivity	Specificity	TBR	Notes
**Barabino** [9]	*88*	*72.4%*	*60.0%*	*NP*	
**Liberale** [10]	*63*	*87.5% non mucinous*	*100% non mucinous*	*1.92 (SD 0.67) for nonmucinous* *0.98 (SD 0.21) for mucinous*	*29% changes in planned operation*
**Lieto** [11]	*69*	*96.9%*	*75%*	*NP*	*Accuracy 95.6%* *25% changes in planned operation*
**Satou** [12]	*10*	*100%*	*100%*	*NP*	
**Tummers** [13]	*21 in 2 pts*	*100%*	*NC*	*2.0*	*FP 62%*
**Veys** [14]	*102*	*72.6%*	*54.2%*	*1.8 (SD 1.3), in scars 2.06 (SD 1.16)*	*PPV 76.8%, in scars 57.1%*

*NP* not provided

## Discussion

This study analyzes the available evidence on the value of ICG fluorescence imaging in enhancing peritoneal visualization of cancer nodules. Many solid abdominal malignancies may cause peritoneal carcinomatosis. Tumor diffusion to the peritoneum represents a systemic cancer extension which, similar to the presence of hematogenous metastases, marks the substantial impossibility of definitively healing the patient. However, it is still possible to cure a selected subgroup of these patients, with results dependent either on biological aspects (for example, peritoneal seeding from ovarian is less aggressive than seeding from pancreatic and stomach tumors), either on stage in which the diagnosis is made [15]. It is therefore very important to detect peritoneal carcinosis at an early stage, allowing to establish the prognosis with greater precision, giving to the patient the correct pathway of care. From the therapeutic point of view, the only possibilities of peritoneal carcinomatosis treatment are linked to a complete surgical reduction, eventually associated with intraperitoneal chemotherapy [16]. Complete cytoreduction is achieved only in presence of limited disease [17]. Unfortunately, the radiological instruments commonly used in the staging of abdominal tumors (CT, MRI, Pet, US) have a poor sensitivity for small peritoneal nodules [2, 3]. The best diagnostic tool is surgical exploration, most frequently by laparoscopic approach, associated with cytological examination on spontaneously present fluid or on peritoneal washing. However, even this technique has a limited sensitivity for peritoneal implants few millimeters-sized [18].

ICG, approved for clinical use by the Food and Drug Administration (FDA) since 1959, is the most commonly utilized fluorescent probe. It is a low-cost molecule, easy to use, widely available and with negligible toxicity [19]. The use of ICG fluorescence in abdominal surgery has been introduced in recent years and represents a common tool for perfusion evaluation, extrahepatic bile duct anatomy, lymph node navigation and liver surgery [20–22]. ICG binds primarily to serum albumin and other serum globulins such as alpha1-lipoprotein, and then it circulates behaving like a macromolecule [23]. In tumor tissues, such as peritoneal cancer implants, an “enhanced permeability and retention” (EPR) effect has been demonstrated, owing to tumor-induced angiogenesis, different metabolic activity and lack of efficient lymphatic drainage [24, 25]. ICG has theorical advantages as a possible contrast agent for macro- and microcirculatory tissue characterization, and consequently for EPR effect: it is uninfluenced by tissue optical properties and has half-life in plasma of few minutes [26, 25]. Some observations seem to indirectly confirm this point. In a series of patients with colorectal cancer, Filippello and Coll. reported that the fluorescence of carcinomatosis nodules was higher, and conversely that the rate of false-negative results was lower, in patients who did not receive bevacizumab compared with those who received the drug (76.3 and 65.0%, 42.9 and 53.8%, respectively). The anti-angiogenetic properties of bevacizumab may attenuate the enhanced permeability and retention of ICG [8]. To date, it is not known whether these theoretical considerations have clinical confirmation. Small nodules size may represent a limiting factor. Furthermore the dosage, and above all the ideal timing of the injection are not clear. Looking at the results, the possible use of this technique compared to simple visual observation in terms of sensitivity, specificity and accuracy has not been clarified.We found only 6 papers, all published in a short period of time (2015–2018) and by a few centers (Saint Etienne, Bruxelles, Naples, Tokyo, and Leiden). Modalities and timing of ICG injection were very similar. After some initial cases, almost all the Authors injected ICG at the time of anesthesia induction and detected fluorescence starting from 5′ after the injection, for a rather long period (someone up to 360′). Some experimental observations suggested that the best timing for ICG visualization due to the EPR is 6 h after injection owing to the rapid clearance of ICG, resulting in a better tumor-to-background ratio starting after 6 h and lasting until 24 h [27]. In case of HCC the timing is different, as ICG is metabolized by normal hepatocytes and unexcreted because of bile ducts alteration, elightening the nodules even many days after the injection. In the case report of peritoneal implant of previously ruptured HCC excluded from the present systematic review, injection of 0,5 mg/kg ICG was performed 72 h before surgery [6]. From a dosing point of view, virtually all series carry the same dosage (0.25 mg/kg), except one that carries twice and one that uses a fixed dose independently from weight (20 mg). All reported injected schedules are similar to that used in other areas for perfusion studies [21].

Overall, in the present systematic review we identified 71 patients. The vast majority (67 cases) were patients with known peritoneal carcinomatosis, undergoing elective surgery for cytoreduction and eventually HIPEC. This clinical setting was deemed ideal for assessing the diagnostic performance of ICG on peritoneal carcinosis, being able to classify each lesion as ICG+/ICG -, malignant/benign by histological examination. By this way, 322 nodules were assessed. Statistical analysis confirmed that ICG may be useful, with sensitivity ranging from 72 to 100% and specificity from 54 and 100%. The average of these values was sensitivity 88.2% and specificity 77.8%. In some papers, subgroups of patients were also investigated. Colorectal carcinomas have been studied in relation to the mucinous component, concluding that this type of cancer has a poor affinity for ICG [10]; furthermore, in series investigating ovarian tumours, the accuracy of ICG on peritoneal scars after neoadjuvant chemotherapy was studied, reporting that this tissue also has little affinity for ICG [14]. Some papers provided quantitative data, calculating the tumor-to-background ratio (TBR) [10, 13, 14]. TBR values around 2.0 have been consistently observed in ICG positive carcinosis nodules. However, in the remaining series, in which TBR has not been calculated, carcinosis nodules have still been detected as fluorescent. Aiming at an immediate and wider clinical spreading of this staging technique in clinical practice, we consider the quantitative aspect not essential.

Only in 4 cases peritoneal carcinosis was not known before surgery (all were open staging of ovarian (3 cases) and uterus cancer (1 case)) [13]. No paper focuses on ICG use during laparoscopic staging of abdominal cancers, with the exception of a single case report [6] and a series mainly focused on small hepatic surface metastases from periampullary cancers [7]. Laparoscopic does actually represent a standard staging and therapeutic approach for many abdominal malignancies [28, 29]. Many laparoscopic vision systems are currently equipped with fluorescence-driven surgery technology, and laparoscopic setting appears even easier than the open one, in which a camera held by the hand, often quite cumbersome, may slowing down the intervention. A monocentric study on pancreatic malignancies has been proposed in 2019 [30]. We personally believe that staging laparoscopy for advanced gastric cancer, which is provided by international guidelines before neoadjuvant or palliative chemotherapy [13] may be also a suitable setting for a prospective randomized study.

In the present systematic review we decided to consider only papers describing the use of ICG to detect nodules of peritoneal carcinosis, with the aim of evaluating the practical effectiveness of this technique in terms of an immediate translation in the daily practice. Some other fluorophores have been studied in humans, but none of them can currently be used outside experimental setting [31–34]. For the same reasons we decided not to consider studies on animal models and in vitro studies [33–35]. Furthermore, we have not considered studies in which ICG is linked to molecules that bind directly to cancer cells. This field of research is very exciting from a theoretical point of view, and many papers have recently reported impressively favourable results; this strategy could make fluorescence a truly oncological navigation [33, 36–38]; however, the need for an advanced molecular biology and biochemistry laboratory, ethical concern requiring IRB approval, high costs, make it still far from a practical application [39].

This study has some limitations. First, and most important, some weakness point in the methodology, should be disclosed: the lack ok meta-analysis and the lack of risk for bias assessment. Secondly, the number of involved centers and the number of analyzed patients are limited. Thirdly, the setting in which this method could be most useful (staging laparoscopy for neoplasms with frequent peritoneal involvement) has never been analyzed in the papers included in this review. Finally, the ideal ICG timing and dosage have not been definitively etablished by dose-findings studies. Further prospective multicenter studies are warranted to establish the actual diagnostic yeld of this method.

## Conclusion

The results of this systematic review highlight the possible role of ICG-fluorescence detection of peritoneal carcinomatosis. Very few clinical data are available, all pertaining to retrospective studies in which this technique was applied during cytoreductive surgery in patients with known peritoneal disease. Only colorectal, hepatocellular and ovarian cancer patients were investigated. In this setting, ICG could diagnose peritoneal nodules with intermediate accuracy, but in some cases it changed the planned intervention. Further prospective studies are warranted, including the setting of staging laparoscopy in patients without radiologically evident peritoneal involvement.

## Data Availability

Data were extracted directly from full-text papers (see references, in particular the 6 case series included in the systematic review are listed as reference [9–14], and are accessible on pub-med.

## Abbreviations

ICG: IndoCyanine Green
HIPEC: Hyperthermic IntraPEritoneal Chemotherapy
TBR: Tumor-to-Background Ratio
CT: Computed Tomography
MRI: Magnetic Resonance Imaging
US: UltraSonography
FDA: Food and Drug Administration
